# Paternity Outcomes in the Freshwater Gastropod, *Chilina dombeiana* in the Biobío River, Chile

**DOI:** 10.1371/journal.pone.0169574

**Published:** 2017-01-09

**Authors:** Jéssica Bórquez, Antonio Brante

**Affiliations:** Departamento de Ecología, Facultad de Ciencias, Centro de Investigación en Biodiversidad y Ambientes Sustentables (CIBAS), Universidad Católica de la Ssma, Concepción, CHILE; Australian Museum, AUSTRALIA

## Abstract

Studying the mating system of obligate aquatic organisms that inhabit river ecosystems is important for understanding its evolution as well as the role of biological and environmental factors in modulating population dynamics and species distributional patterns. Here, we studied the reproductive strategy of the Chilean endemic freshwater snail, *Chilina dombeiana*, in the Biobío River, one of the largest rivers in Chile. This species has a low potential for dispersal given the absence of a free-swimming larval stage (benthic larval development) and given that adults have a low capacity for mobility. We hypothesized that: 1. Females would mate with different males (polyandry) resulting in intrabrood multiple paternity, 2. Individuals from closer sites would be more related than individuals from distant sites, and 3. Male parental contributions would be unevenly distributed within broods. Individuals from three different sites were sampled along the river: upper, mid, and river mouth. In the laboratory, hatching juveniles from a total of 15 broods were collected for paternity analyses. We used microsatellite markers and the programs GERUD and COLONY to determine whether multiple paternity exists and to estimate the contribution of different males to the brood. We found that multiple paternity was very common at all of the sites analyzed with as many as 8 males fertilizing a single female and a mean of 4.2 fathers per brood estimated by COLONY. Sire contribution was skewed to particular males in several broods. In addition, overall relatedness among broods for the three sites ranged from 0.17 to 0.45 with evidence of many half-siblings. Relatedness differed among the three sites. Particularly in upstream sites or in anthropogenically disturbed populations, the high levels of multiple paternity observed in *C*. *dombeiana* may be an efficient strategy to avoid inbreeding and prevent the loss of genetic diversity within populations.

## Introduction

Polyandry, the act of a female mating with multiple males, is one of the most common mating systems observed in nature and has been reported in many taxonomic groups [1,2,3,4,5,6,7,8]. Often, polyandry results in multiple paternity with females laying eggs fertilized by different males, and male contribution to the brood occurs in different proportions [9]. According to the literature, there are individual costs and benefits to polyandry; for example, costs include increased exposure to sexually transmitted disease [10], increased risk of predation [11,12,13], and potential effects on foraging behavior [14]. In contrast, due to the greater supply and increased quality of sperm [15,16] resulting from polyandry, potential benefits could include increased fertilization success [14] and prevention of the deleterious effects of inbreeding depression [17,15]. In addition, higher genetic variability among siblings within a brood has been observed as a result of polyandry; thus, offspring could have a higher evolutionary potential to respond to environmental changes [18,15,19].

The main difficulty in evaluating the effect of polyandry on fitness, especially in males, is obtaining reliable estimates of reproductive success. Various pre- and post-fertilization mechanisms occur that may affect paternal contributions, such as cryptic female choice, sperm viability, sperm competition, sperm-ovum compatibility, and sibling cannibalism [20,21]. Fortunately, genetic markers such as DNA microsatellites are powerful tools for studying mating systems and for measuring paternity. Due to the high variability, neutrality, codominance, and Mendelian inheritance of these types of markers, microsatellites can be used to construct pedigrees and reliably infer demographic gene flow [22,1].

For invertebrate species, most of our knowledge of mating systems and reproductive behavior comes from genetic studies of reproductive success in terrestrial and marine systems; far less is known for the freshwater realm [23]. In contrast to other aquatic systems, river ecosystems have a hierarchical structure of dendritic stream networks, which can affect the pattern of dispersal and connectivity between populations that are usually dominated by unidirectional migration [24]. Also, rivers are subjected to high anthropogenic pressure including habitat loss, habitat restructuring and fragmentation; this, in turn, may reduce the likelihood of encountering a mate, increase inbreeding, decrease genetic diversity, and may promote local extinction [22,25,26]. In this way, it is expected that multiple paternity through intense polyandry may enhance the local viability of populations inhabiting river ecosystems. However, the molecular evidence for multiple paternity occurring in invertebrate species from these habitats is scarce. The few reports of freshwater invertebrates are biased towards those of mussel sperm release (*Lampsilis cardium*, [27]; *Villosa iris*, [28]; *Hyriopsis cumingii*, [29]). One of the few studies in existence based on genetic data reports multiple paternity yet high evenness of sire fertilization for two populations of the freshwater snail *Potamopyrgus antipodarum* [23].

*Chilina dombeiana* (Bruguière, 1789) is native to Chile and belongs to an ancient family of freshwater gastropods, Chilinidae Dall 1870 (Gastropoda, Hygrophila). This family has a single genus, *Chilina* Gray 1828, and is found only in South America [30]. *C*. *dombeiana* exhibits simultaneous hermaphroditism and although there is no evidence, this species like other gastropods from the order Hygrophila, could be capable of self-fertilization and/or biparentality through cross-fertilization [30,31,32]. *C*. *dombeiana* is oviparous with internal fertilization and direct development (absence of a free-swimming larval stage). The eggs are encapsulated and embedded in gelatinous zig-zag-like string egg masses that are usually attached to rocky substrates [33]. *C*. *dombeiana* is widely distributed in Chilean river ecosystems between 35° and 41° S and has high population densities. It is common to observe aggregations of individuals on or near patchily distributed rocky platforms and boulders (pers. obs.). Furthermore, adults have been observed to aggregate during reproductive periods and when laying of egg masses occurs (pers. obs.). In spite of their high abundance and wide distribution, there is scarce information on the biology and ecology of this organism and even less is known of the mating system and reproductive strategy of *C*. *dombeiana*.

Here we studied *C*. *dombeiana* populations inhabiting the Biobío River, the third largest river basin in Chile [34]. The Biobío River is a fragmented habitat that has been subjected to a great deal of anthropogenic disturbance given its proximity to urban centers [35]. The physical characteristics of the habitat together with the direct development of this species and its sedentary behavior could result in high population structuring and high inbreeding. Using microsatellite markers, we studied the mating system of *C*. *dombeiana* in three sites along the Biobío River. We expect to find: 1. multiple paternity in this species, 2. higher relatedness between individuals of closer sites than between individuals of distant sites, and 3. uneven male parental contribution within broods.

## Materials and Methods

### Ethics statement

Handling of *C*. *dombeiana* individuals was conducted in accordance with the biosafety and bioethics guidelines of the National Commission for Scientific and Technological Research (CONICYT) of Chile. No conservation or management policies are considered for *C*. *dombeiana* and the study was not conducted in protected areas, thus no specific permission was required for sampling individuals.

### Sampling

During December 2015, we collected by hand a total of 60 adult *C*. *dombeiana* individuals from three different sites (20 individuals from each site) along the Biobío River, Biobío Region, Chile. The three sites included the upper Biobío (UBB; 37°40´S, 72°0´W), the mid Biobío near to the locality of Negrete (MBB; 37°35´S, 72°28´W), and close to the mouth of the Biobío River (MoBB; 36°49´S, 73°6´W). After collection, specimens were individually stored in 95% ethanol in plastic tubes until later genetic analyses were conducted.

For parentage analysis, a total of 15 *C*. *dombeiana* adults, five from each site, with shell lengths ranging from 15 to 22 mm were transported alive in a cooler filled with freshwater to the Faculty of Science, Universidad Católica de la Santísima Concepción. In the laboratory, adults were placed in individual plastic boxes (200 ml) filled with freshwater collected from the sample sites. A small rock was added to each box to serve as substrate to stimulate egg mass laying. The boxes were constantly aerated with an air pump, and a 12 h light/dark photoperiod and constant temperature of 18°C were maintained during the entire experimental period. Additionally, the water inside the boxes was changed every ten days. After approximately one week, adult individuals (named *females* here after) laid egg masses with lengths ranging from 30 to 70 mm. From each egg mass, 21 pre-hatching juvenile individuals (approximately 25% of the total brood) were randomly chosen for paternity analysis. The females (mothers) and 315 juvenile offspring were placed in individual tubes and stored in 95% ethanol.

### DNA extraction and microsatellite genotyping

DNA extraction of the juveniles was conducted using the whole animal, meanwhile for adult individuals about 3 mm^3^ of muscle tissue was taken from the foot. DNA extraction was performed using the Thermo Scientific Direct PCR kit following the manufacturer’s instructions. The template genomic DNA was diluted to 10ng/μl. Genotyping of microsatellites was conducted using primers fluorescently labeled with 6FAM, VIC, PET or NED. Developed by Genetic Marker Services, eleven variable microsatellite loci were successfully amplified in *Chilina dombeiana* (nine of these were previously reported [36]) and were used for paternity analyses. A total of 20 ng of genomic DNA was used as the template for polymerase chain reactions (PCR). The 10 ul reaction volumes contained: 1.5 mM MgCl_2_, 1X buffer, 200 μM of each dNTP, 3 pmol of each primer, and 0.25 U of Go Taq Hot Start polymerase (Promega). Amplification was conducted in a thermal cycler (Verity) with the following cycling parameters: 35 to 40 cycles for 60 s at 95°C, 60 s at 55°C and 30 s at 72°C. An initial denaturing step (95°C, 2 min) was included and the last cycle was followed by a 10 min extension at 72°C. The PCR products were separated on an ABI3730 XL automated DNA sequencer with the LIZ-500 size standard (Applied Biosystems); this was conducted in the Genomic Facility Center of University of Guelph (Canada). The allele sizes for each female and for the offspring were analyzed using the program GeneMarker v2.2 (Softgenetics, State College, PA, USA).

### Statistical and parentage analyses

For each site the assumptions of Hardy–Weinberg and linkage equilibrium were tested in GENEPOP 4.1 [37], and significance levels were adjusted using the sequential Bonferroni correction for multiple comparisons [38]. Loci were checked for the presence of null alleles in MICROCHECKER V.2.2.3 [39]. The expected (He) and observed (Ho) heterozygosities were calculated using the program GENETIX [40], inbreeding coefficient (Fis) and average allelic richness over all loci (AR) were estimated using the program FSTAT [41].

Before paternity analyses, the probability of exclusion (PE), which considers the probability to discard as a true parent an unrelated individual, was estimated over all loci using GERUD 2.0 [42]. PE was estimated for each site taking into account the allelic frequency of adults from that site and considering that one parent (the mother) was known with certainty. Then, we conducted a power analysis for each site to test if the set of seven loci and 21 pre-hatching individuals per clutch sampled in this study were adequate to detect multiple paternity. For this, simulation analyses implemented in the software PrDM [43] were run for scenarios of equal, moderately skewed, and highly skewed paternal contribution taking into account a varying number (two to ten) of participant males in a brood. This range of participant males is in accordance with the level of multiple paternity observed in other aquatic gastropods [44,45].

The analysis of paternity was conducted using two different softwares GERUD 2.0 [42] and COLONY [46,47]. GERUD is a computer program that estimates the minimum number of fathers even when the father genotypes are unknown. GERUD starts by subtracting the known maternal genotype and then determines the minimum number of sires that can explain the entire progeny array. If multiple possible genotypes exist, the program proposes the most likely solution based on the laws of Mendelian segregation and based on the allele frequencies in the population. The program assumes that all offspring are full or half-siblings, which is a requirement according to the internal fertilization of *C*. *dombeiana*. Six to eleven microsatellite loci were used in the analysis; in some cases loci with missing data were discarded. COLONY is another computer program that uses mathematical algorithms to determine the number of sires when paternal genotypes remain unknown. While GERUD estimates the minimum number of sires, COLONY estimates the most likely number of sires. Thus, COLONY was used to estimate the maximum number of sires per brood using a maximum-likelihood method to assign parentage and sibship groups; from this, the genotypes of the unsampled parents were also reconstructed [48]. Three replicate analyses were conducted for each family. ‘Medium’ length and ‘medium’ likelihood precision runs employing adult allele frequencies, different random number seeds, and genotyping error rate of 0.01were used for the analysis.

In order to investigate relatedness of offspring within families and whether or not individuals from families within a site were more related compared to individuals from other families and sites, we computed and compared pairwise relatedness estimator values (r) using COANCESTRY 1.0.1.5 [49]. Pairwise relatedness for each site was calculated using three estimators, the Queller and Goodnight (1989) [50], Lynch and Ritland (1999) [51], and Wang (2002) [52] estimators. Because all estimators gave similar results, only the Wang estimator results are presented. Resulting r values may range between -1 and 1, with negative values indicating that individuals share fewer alleles than average. Theoretical r values in a randomly mating population are 0.5 for full siblings and 0.25 for half-siblings. Additionally, using COANCESTRY’s bootstrap estimator method, we tested for significant differences in mean pairwise relatedness between sites; two-tailed tests included 10,000 bootstraps [49].

To determine if the reproductive contribution of different fathers in the multiple-sired broods differed significantly from the expectation of equality (evidence of significant reproductive skew), we calculated sire evenness (E). This metric was estimated using an index of sire diversity D, based on the Shannon-Wiener index (following [23]). Sire diversity D was calculated by:

$$D=\;-\;\overset{}{\underset{}{\Sigma_{i=1}^{S}}}p_{i}\;\;\times \;ln\;p_{i}\;$$

In this formula, *S* equals the number of genetic sires in a brood and *p*_*i*_ equals the proportion of offspring sired by the *i*^th^ sire. Once *D* was obtained, evenness *E* could be calculated by utilizing an additional index, *D*_*max*_. This index is a hypothetical diversity score where *D*_*max*_ = ln *S*; thus, this value is the maximum possible sire diversity if offspring had been distributed evenly given the number of genetic sires. Whether or not a female has reached maximum diversity within the clutch is limited to a set number of sires (evenness). This is calculated by (following [23]):

$$E=\frac{-\sum_{i=1}^{S}\;p_{i}\;\;\times \;ln\;p_{i}}{lnS}=\;\frac{-\sum_{i=1}^{S}\;p_{i}\;\;\times \;ln\;p_{i}}{Dmax}$$

To examine whether paternal contributions deviated significantly from equality in each brood, goodness of fit χ^2^ tests with the null hypothesis of equal reproductive contribution were made in R 2.13.1 [53].

Given that *C*. *dombeiana* is a simultaneous hermaphrodite we explored the potential existence of self-fertilization. The classical approach of measuring heterozygote deficiencies (*F*_IS_) has been used widely to estimate inbreeding, but this method is associated with typing artefacts, null alleles, and allele drop-out among other common problems that have been discussed in the literature [54]. As such, we used the multilocus structure of the dataset to compare the distribution of multiple-locus heterozygosity and identity disequilibria. Thus, we were able to provide robust estimates of selfing rates given our microsatellite data (methods followed the study of David et al. 2007) [54]. Selfing rate (s) was estimated from maximizing the log-likelihood of the multilocus heterozygosity structure (ML) and two-locus heterozygosity disequilibrium (g2). For (s) estimates we used the R package “inbreedR” [53] and software RMES [54].

## Results

### Genetic variability of adults

The set of eleven microsatellite loci are characterized for each site in Table 1. For the adult dataset, the mean allelic richness over all loci (AR) was 4.75 (range: 4.27–5.90) and observed heterozygosity (Ho) ranged from 0.42 to 0.52. Loci were in Hardy-Weinberg equilibrium in all sites (Table 1). There was no apparent pattern of deviations from gametic disequilibrium between marker pairs for any loci or sites. Tests in Micro-Checker did not provide any evidence for the presence of null alleles at any locus. Significant levels of the inbreeding coefficient (F_IS_) were determined for each site; F_IS_ values ranged from 0.063 to 0.092 (Table 1).

**Table 1:** Summary statistics of genetic variability for three sites along the Biobío River: Upper Biobío (UBB), Mid Biobío (MBB), and mouth of the river (MoBB).

Sites	AR	He	Ho	P(0.95)	P(0.99)	F_IS_	HWE	s(g_2_)	P_g2 = 0_	s(ML)	CI(95)
UBB	4.90	0.557	0.52	0.909	0.909	0.092	0.120	0.018	0.449	0	[0, 0.148]
MBB	5.90	0.490	0.46	0.909	1	0.089	0.229	0	0.553	0.071	[0, 0.274]
MoBB	4.27	0.439	0.42	0.909	0.909	0.063	0.196	0.086	0.189	0.111	[0, 0.292]

A total of 20 adult individuals were analyzed for each site. Expected (He) and observed (Ho) heterozygosity, proportion of polymorphic loci (P) with the commonest allele present at no more than 95 and 99%, average allelic richness over all loci (AR), inbreeding coefficient (F_IS_) and probability of deviation from Hardy-Weinberg equilibrium (HWE) are indicated for the eleven polymorphic microsatellite loci used in this study. Estimations of the selfing rate (s) are shown for the two methods implemented: s(g_2_) and maximum likelihood (s(ML)). Power to reject the null hypothesis s = 0 at p<0.05 (P_g2 = 0_), and the 95% confidence intervals (CI) for the ML method are also included.

The combined probability of exclusion for all of the eleven microsatellite markers was over 0.98 for the three sites analyzed. Considering the number of loci, number of prehatching juvenile snails sampled for paternity analyses, and the knowledge of the mothers’ genotypes, results from the PrDM simulations indicated a high probability of detecting multipaternity involving a range of two to ten fathers; statistical power provided by a minimum of seven microsatellite markers ranged from 83% to 100% (Table 2). Estimations of (s) showed that the probability of self-fertilization in this species in the three sites sampled was not significantly different from 0 (Table 1). These results were consistent for both methods used (ML and g2).

**Table 2:** The probability of detecting multiple paternity (PrDM) for different mating scenarios (from two to ten males) for the three sites along the Biobío River: Upper Biobío (UBB), Mid Biobío (MBB), and mouth of the river (MoBB). This was determined by PrDM simulations involving 21 progeny sampled per clutch and a minimum of seven microsatellite markers. The simulated mating scenarios were based on the minimum and maximum number of sires reported by other studies of aquatic gastropods, and the relative male contribution (equal, moderately skewed, or highly skewed) of males to the brood was varied.

Mating scenario	Relative male contributions (%)	PrDM		
		UBB	MBB	MoBB
2 males	(50:50)	0.999	0.997	0.990
	(75:25)	0.98	0.989	0.979
	(90:10)	0.862	0.850	0.829
3 males	(33:33:34)	1	1	1
	(50:25:25)	1	1	1
	(80:10:10)	0.985	0.982	0.975
7 males	(14:14:14:14:14:15:15)	1	1	1
	(30:20:15:10:10:10:5)	1	1	1
	(50:30:10:5:2:2:1)	1	1	1
10 males	(10:10:10:10:10:10:10:10:10:10)	1	1	1
	(30:10:10:10:10:10:5:5:5:5)	1	1	1
	(50:20:10:5:5:3:2:2:2:1)	1	1	1

### Paternity

Fathers were assigned to 315 offspring for which the mother was known. Using both GERUD and COLONY, the results revealed evidence of high levels of multiple paternity in all families across all sites analyzed (Table 3).

**Table 3:** Paternity inference (number of sires within broods) conducted in GERUD and COLONY. Pairwise relatedness (r) and mean pairwise difference in relatedness between sites were estimated in COLONY. Sire evenness within broods estimated from paternity assignments carried out in GERUD and COLONY are also shown.

Family	Female	Loci‡	Paternityⱡ	Pairwise relatedness (r)		Difference mean(±95%)+	Sire Evenness*	
	ID			Mean	(±SD)		GERUD	COLONY
F1	UBB1	9/ 8	3/8	0.201	0.141		0.94	0.95
F2	UBB2	10/ 8	2/2	0.451	0.006		**0.45**	**0.59**
F3	UBB3	10/7	4/4	0.297	0.075		0.96	0.99
F4	UBB4	9/8	3/4	0.173	0.128		**0.81**	0.99
F5	UBB5	8/8	3/4	0.311	0.064		0.85	0.93
			**Site UBB**	**0.287**	**0.109**	1.072E002^a^		
F6	MBB1	10/9	3/5	0.274	0.055	(-1.038E002-1.052E002)	**0.81**	0.93
F7	MBB2	11/8	3/3	0.410	0.071		**0.79**	0.90
F8	MBB3	10/9	2/3	0.372	0.082		**0.59**	**0.83**
F9	MBB4	10/9	4/4	0.251	0.133		0.85	0.85
F10	MBB5	11/8	3/3	0.377	0.05		0.90	0.99
			**Site MBB**	**0.337**	**0.069**	6.550E002^b^		
F11	MoBB1	9/9	4/7	0.409	0.081	(-1.069E002-1.030E002)	**0.81**	0.92
F12	MoBB2	10/10	3/4	0.297	0.088		0.98	0.91
F13	MoBB3	11/8	4/5	0.261	0.066		**0.86**	0.97
F14	MoBB4	11/10	3/3	0.295	0.111		0.92	**0.84**
F15	MoBB5	10/10	2/4	0.379	0.075		**0.81**	**0.64**
			**Site MoBB**	**0.328**	**0.062**	7.623E002^c^		
						(-1.077E002-1.084E002)		
Mean			3.06/4.2					

^‡^ N°loci: number of polymorphic microsatellite loci used in COLONY/GERUD respectively

^ⱡ^ number of sires calculated in GERUD/COLONY.

^+^ mean pairwise differences in relatedness between sites were estimated by bootstrap analysis in COLONY: (a) = Sites UBB and MBB, (b) = Sites MBB and MoBB, (c) = Sites UBB and MoBB

*values in bold are significant (P<0.05).

**Fig 1:**
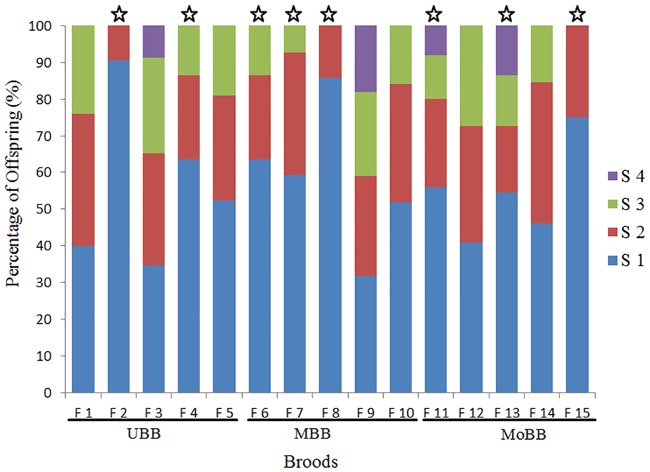
The relative contribution of sires to *Chilina dombeiana* broods from three sites of the Biobío River: the upper site (UBB), Mid site (MBB), and the mouth of the river site (MoBB). The minimum number of fathers was estimated in GERUD. The colors within the bars depict the relative contribution of different sires to the broods (S1-S4). Stars above the bars indicate that paternal contributions within a brood deviated significantly from equality (goodness of fit X^2^ tests, *P* < 0.05).

**Fig 2:**
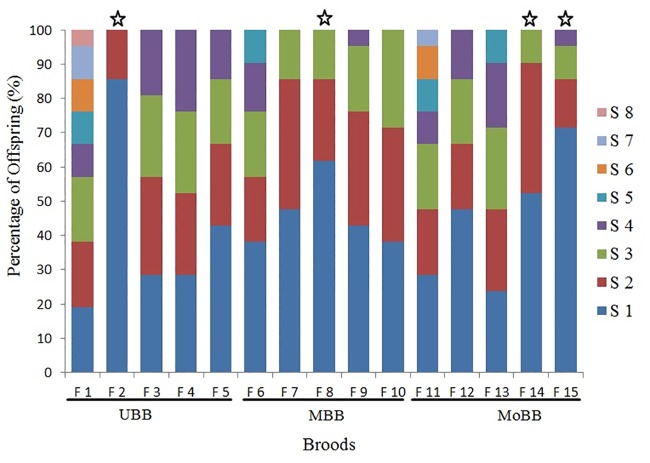
The relative contribution of sires to *Chilina dombeiana* broods from three sites of the Biobío River: the upper site (UBB), Mid site (MBB), and at the mouth of the river site (MoBB). The maximum number of fathers was estimated in COLONY. The colors within the bars depict the relative contribution of different sires to the broods (S1-S8). Stars above the bars indicate that paternal contributions within a brood deviated significantly from equality (goodness of fit X^2^ tests, *P* < 0.05).

The minimum number of sires estimated in GERUD ranged from two to four with an average of 3.06 sires per family (Table 3, Fig 1). Using COLONY, an average of 4.2 maximum sires were estimated per family (Table 1, Fig 1). Considering sites individually, 2–4 sires were estimated for clutches from the upper Biobío river (UBB), Mid (MBB) and the mouth (MoBB) of the Biobío river (GERUD results). The maximun number of fathers estimated in COLONY ranged from 2 to 8 for site UBB, 3 to 5 for site MBB, and between 3 and 7 for MoBB (Table 3, Fig 2).

Sire eveness estimated from male contributions using GERUD ranged from 0.45 to 0.96 for the UBB site, 0.59 to 0.90 for the MBB site, and 0.81 to 0.98 for the MoBB site (Table 3). Using COLONY, sire eveness was variable between broods and ranged from 0.59 to 0.99 for the UBB site, from 0.83 to 0.99 for the MBB site, and from 0.64 to 0.97 for the MoBB site (Table 3). Sire eveness calculated from GERUD deviated significantly from equality in 8 of the 15 families analysed (goodness of fit X^2^ test, *P < 0*.*05*; Table 3). In the other families, three from the UBB site and two from the MBB and MoBB sites, the proportion of fertilization by multiple sires was equally distributed (Table 3, Fig 1). Estimations of sire evenness from COLONY showed that four of the 15 families showed significant deviations from equality (goodness of fit X^2^ test, *P < 0*.*05*; Table 2). In the remaining families, four from UBB and MBB sites and three from the MoBB site, the proportion of fertilization by multiple sires was equally distributed (Table 3, Fig 2).

Mean relatedness level within broods was 0.287 (±0.109), 0.337 (±0.069), 0.328 (±0.062) for sites UBB, MBB, and MoBB, respectively (Table 3). Overall relatedness among broods for the three sites ranged from 0.17 to 0.45; this suggests that half-siblings make up a large proportion of the population (around 80%; Table 3). The distribution of relatedness across sites tested by pairwise comparisons in COANCESTRY (bootstrapping:10.000) revealed significant differences among the three sites (95% confidence level; Table 3).

## Discussion

Multiple paternity has been frequently observed in marine gastropod mollusks, however little is known about paternity in freshwater snails. In fact, no study to date has investigated patterns of multiple paternity in pulmonate Chilinidae species and in particular, in the endemic Chilean species, *Chilina dombeiana*. The present study provides the first insights into the occurrence and frequency of multiple paternity in this species. Multiple paternity was detected in all 15 families analyzed, using two different programs (GERUD [42] and COLONY [47]), originating from three different sites along the Biobío River, Chile. Mean relatedness differed between sites and overall estimates of relatedness across families showed evidence for high levels of half-siblings. No significant evidence of self-fertilization was obtained.

Freshwater snails are simultaneous hermaphrodites and most species preferentially self-fertilize or outcross [55,56,22,57]. For example, a study of the freshwater snail, *Bulinus truncatus*, has revealed that this gastropod mainly self-fertilizes [58]. In contrast, the tropical freshwater pulmonate *Biomphalaria glabrata* primarily outcrosses [57]. Several species potentially have mixed mating strategies. For example, the invasive freshwater snail *Physa acuta* can self-fertilize or outcross, but outcrossing is the main mating system in populations of this species [59,60]. In the present study, the selfing rates of *C*. *dombeiana* were not significantly different than 0 in the three sites, and this was seen across the two different methods tested. This suggests that self-fertilization in this species is either very rare or may not occur.

Multiple paternity has been frequently observed in marine gastropod mollusks, but to date there are few studies documenting this in freshwater systems (e.g. marine species: [61,45,3,4,5]; freshwater species: [23]). Several decades ago, studies using enzyme electrophoresis reported multiple paternity in freshwater pulmonates such as *Biomphalaria obstructa* [62], *Bulinus africanus* [63], *B*. *cernicus* [64], *Physa heterostropha pomilia* [65], *Ancylus fluviatilis* [66] and *Lymnaea peregra* [67]. However, the low resolution of enzyme analyses precludes a more detailed description of the reproductive strategy of these species. In the last decade, highly variable nuclear microsatellite markers have solved these limitations and have been widely used for parentage analysis. Despite this, only few parentage studies have been carried out in freshwater snails. One of the a few examples has shown that females of the freshwater caenogastropod *Viviparus ater* mate with different males and may store sperm [68]. In the New Zealand freshwater snail *Potamopyrgus antipodarum*, multiple paternity ranged from two to four sires as detected using GERUD and between four and seven sires when estimates were made using COLONY. Additionally for *P*. *antipodarum*, sires contributed evenly within the broods [23]. However, only three microsatellite markers were used in the study of the New Zealand snail, thus an expanded polymorphic dataset would allow for more exact estimations of sire number and evenness [23].

In the present study, paternity analyses revealed sperm storage and high levels of multiple paternity within broods of *C*. *dombeiana*. As estimated in GERUD, a minimum of two to four sires participated in each clutch, and a maximum of 2 to 8 participating males were estimated in COLONY. No major differences in the number of sires were found between sites. Alternatively, the pattern of sire contribution within broods varied between females and between sites. Using GERUD estimations, paternity was unevenly distributed between males in close to half of all of the broods (47%). Meanwhile, 20% of the broods had unequal male contributions when estimates were made using COLONY. Inter-mating period and mating order [69], sperm storage [3], and the amount of sperm transferred by males to females [70] could be influencing the distribution of paternity in this gastropod.

Mating with multiple individuals can be a costly behavior [71], however it is widespread in nature [72]. The polyandry and multiple paternity of *C*. *dombeiana* may provide benefits to this species in terms of ensuring fertilization and increasing mean offspring genetic diversity, as has been suggested for other invertebrates [73,74]. For example, multiple mating in the hermaphroditic land snail *Cornu aspersum* has been shown to aid fertilization of eggs [69], and multiple mating in the freshwater snail *Potamopyrgus antipodarum* has been thought to increase genetic diversity within broods [23]. In addition, post-copulatory selection may occur via sperm competition. In turn, this may reduce the potential for inbreeding as has been suggested for the freshwater shrimp *Caridina ensifera blue* [75]. Furthermore, post-copulatory selection may increase the quality of the offspring based on the higher viability of offspring from specific male-female crosses [76,77].

The Biobío River is exposed to high levels of anthropogenic pressure in the way of hydroelectric dams, industrial waste, industrial and agricultural water consumption, irrigation runoff, sand extraction, and recreational use [35]. Such perturbations may affect populations of *C*. *dombeiana*, impacting the abundance of this species and causing severe local bottlenecks or occasional local extinctions. Furthermore, the potential effects of anthropogenic stress could be more critical given the absence of dispersal larvae and the sedentary behavior of *C*. *dombeiana* adults. As such, this species could be particularly vulnerable to contamination, habitat fragmentation, and habitat loss [78,34,79]. Should populations of this species become threatened, polyandry in *C*. *dombeiana* could help to ensure fertilization success, could increase genetic diversity, and could reduce the adverse effects of inbreeding. For example, in the marine snail *Littorina saxatilis* it has been suggested that high multiple paternity could potentially increase effective population size when populations experience strong bottlenecks [2].

In summary, the *C*. *dombeiana* shows high level of multiple paternity suggesting that populations of this species may be highly genetically diverse; thus, this species may have the evolutionary potential necessary to respond to changes in environmental conditions and natural and anthropogenic perturbations. Different aspects of the ecology and biology of *C*. *dombeiana* are still unknown; hence additional studies, such as those measuring spatial demography, mating behavior, and anatomical reproductive features are required to explain the paternity patterns observed for this species.

## References

[1] Paterson IG, Partridge V, Buckland-Nicks J. Multiple paternity in *Littorina obtusata* (Gastropoda, Littorinidae) revealed by microsatellite analyses. Biol Bull. 2001; 200: 261–267. 10.2307/1543508 11441969

[2] Panova M, Boström J, Hofving T, Areskoug T, Eriksson A, Mehlig B, et al Extreme female promiscuity in a non-social invertebrate species. PLoS One. 2010; 5:e9640 10.1371/journal.pone.0009640 20300171 PMC2836369

[3] Brante A, Fernández M, Viard F. Microsatellite evidence for sperm storage and multiple paternity in the marine gastropod *Crepidula coquimbensis*. J Exp Mar Biol Ecol. 2011; 396: 83–88.

[4] Lombardo RC, Takeshita F, Ábe S, Goshima S. Mate choice by males and paternity distribution in offspring of triple-mated females in *Neptunea arthritica* (Gastropoda:Buccinidae). J Molluscan Stud. 2012; 78: 283–289.

[5] Xue D, Zhang T, Liu J-X. Microsatellite evidence for high frequency of multiple paternity in the marine gastropod *Rapana venosa*. PLoS One. 2014; 9: e86508 10.1371/journal.pone.0086508 24466127 PMC3900555

[6] Klemme I, Ylönen H, Eccard A. Long-term fitness benefits of polyandry in a small mammal, the bank vole *Clethrionomys glareolus*. Proc R Soc Lond Biol Sci. 2008; 275: 1095–1100. 10.1098/rspb.2008.0038 PMC2600916 18270151

[7] Firman RC, Simmons LW. The frequency of multiple paternity predicts variation in testes size among island populations of house mice. J Evol Biol. 2008; 21: 1524–1533. 10.1111/j.1420-9101.2008.01612.x 18811664

[8] Bergeron P, Réalet D, Humphries MM, Garant D. Evidence of multiple paternity and mate selection for inbreeding avoidance in wild eastern chipmunks. J Evol Biol. 2011; 24: 1685–1694. 10.1111/j.1420-9101.2011.02294.x 21585586

[9] Dunn PO, Lifjeld JT, Whittingham LA. Multiple paternity and offspring quality in tree swallows. Behav Ecol Sociobiol. 2009; 63: 911–922.

[10] Parker GA, Birkhead TR. Polyandry: the history of a revolution. Philos Trans R Soc Lond B Biol Sci. 2013; 368: 20120335 10.1098/rstb.2012.0335 23339245 PMC3576588

[11] Thrall PH, Antonovics J, Bever JD. Sexual transmission of disease and host mating systems: within season reproductive success. Am Nat. 1997; 149: 485–506.

[12] Jennions MD, Petrie M. Variation in mate choice and mating preferences: a review of causes and consequences. Biol Rev Camb Philos Soc. 1997; 72: 283–327. 9155244 10.1017/s0006323196005014

[13] Ashby B, Gupta S. Sexually transmitted infections in polygamous mating systems. Philos Trans R Soc Lond B Biol Sci. 2013; 368: 20120048 10.1098/rstb.2012.0048 23339239 PMC3576582

[14] Hedrick PW. Genetics of populations. 4^th^ edition Boston (MA): Jones and Barlett, Publishers, LLC; 2011.

[15] Slatyer RA, Mautz BS, Backwell PRY, Jennions MD. Estimating genetic benefits of polyandry from experimental studies: a meta-analysis. Biol Rev. 2012; 87: 1–33. 10.1111/j.1469-185X.2011.00182.x 21545390

[16] Kimura K, Chiba S. Strategic ejaculation in simultaneously hermaphroditic land snails: more sperm into virgin mates. BMC Evol Biol. 2013; 13: 264 Available: http://www.biomedcentral.com/1471-2148/13/264 10.1186/1471-2148-13-264 24304518 PMC4235035

[17] Bretman A, Newcombe D, Tregenza T. Promiscuous females avoid inbreeding by controlling sperm storage. Mol Ecol. 2009; 18: 3340–3345. 10.1111/j.1365-294X.2009.04301.x 19694961

[18] Newcomer SD, Zeh JA, Zeh DW. Genetic benefits enhance the reproductive success of polyandrous females. Proc Natl Acad Sci USA. 1999; 96: 10236–10241. 10468592 10.1073/pnas.96.18.10236 PMC17872

[19] Aguirre JD, Marshall DJ. Does genetic diversity reduce sibling competition?. Evolution. 2012; 66: 94–102. 10.1111/j.1558-5646.2011.01413.x 22220867

[20] Eberhard WG, Cordero C. Sexual selection by cryptic female choice on male seminal products, a new bridge between sexual selection and reproductive physiology. Trends Ecol Evol. 1995; 10: 493–496. 21237123 10.1016/s0169-5347(00)89205-8

[21] Brante A, Fernández M, Viard F. Non-random sibling cannibalism in the marine gastropod *Crepidula coquimbensis*. PLoS ONE. 2013; 8: e67050 10.1371/journal.pone.0067050 23805291 PMC3689673

[22] Städler T, Jarne P. Population biology, genetic structure, and mating system parameters in freshwater snails In: Streit B, Städler T, Lively CM, editors. Evolutionary ecology of freshwater animals. Birhäuser, Basel; 1997 pp: 231–262.

[23] Soper DM, Delph L, Lively CM. Multiple paternity in the freshwater snail, *Potamopyrgus antipodarum*. Ecol Evol. 2012; 2: 3179–3185. 10.1002/ece3.408 23301182 PMC3539010

[24] Takahasi Y. Characteristics of River systems In: Dooge JCI, editor. Fresh surface water. Vol I. EOLSS Publishers/UNESCO; 2009 pp. 259–298.

[25] Frankham R, Ballou JD, Briscoe DA. Introduction to Conservation Genetics. 2^nd^ ed Cambridge University Press; 2010.

[26] Wasserman TN, Cushman SA, Shirk AS, Landguth EL, Littell JS. Simulating the effects of climate change on population connectivity of American marten (*Martes americana*) in the northern rocky mountains, USA. Lands Ecol. 2012; 27: 211–225.

[27] Ferguson CD, Blum MJ, Raymer ML, Eackles MS, Krane DE. Population structure, multiple paternity, and long-distance transport of spermatozoa in the freshwater mussel *Lampsilis cardium* (Bivalvia: Unionidae). Freshw Sci. 2013; 32: 267–282.

[28] Christian AD, Monroe EM, Asher AM, Loutsch JM, Berg DJ. Methods of DNA extraction and PCR amplification for individual freshwater mussel (Bivalvia: Unionidae) glochidia, with the first report of multiple paternity in these organisms. Mol Ecol Notes. 2007; 7: 570–573.

[29] Bai Z, Luo M, Zhu W, Lin J, Wang G, Li J. Multiple paternity in the freshwater Pearl Mussel *Hyriopsis cumingii* (LEA, 1852). J Molluscan Stud. 2011; 0: 1–5.

[30] Jarne P, Pointier JP, David P, Koene JM. Basommatophoran gastropods In: Córdoba-Aguilar A, Leonard JL, editors. The evolution of primary sexual characters in animals. Oxford University Press, New York; 2010 pp. 173–196.

[31] Escobar JS, Auld JR, Correa AC, Alonso JM, Bony YK, Coutellec MA, Koene JM, Pointier JP, Jarne P, David P. Patterns of mating-system evolution in hermaphroditic animals: Correlations among selfing rate, inbreeding depression and the timing of reproduction. Evolution. 2011; 65: 1233–1253. 10.1111/j.1558-5646.2011.01218.x 21521187

[32] Nakadera Y, Swart EM, Maas JPA, Montagne-Wajer K, Maat AT, Koene JM. Effects of age, size, and mating history on sex role decision of a simultaneous hermaphrodite. Behav Ecol. 2014; 26: 232–241. 10.1093/beheco/aru184 25713474 PMC4309981

[33] Bórquez J, Valdovinos C, Brante A. Intracapsular development in the freshwater gastropod *Chilina dombeiana* (Bruguière, 1789) (Gastropoda: Hygrophila: Chilinidae). Nautilus. 2015; 129: 169–171.

[34] Valdovinos C. Estado de conocimiento de los gastrópodos dulceacuícolas de Chile. Gayana 2006; 70: 88–95.

[35] Valdovinos C, Parra O. La Cuenca del Río Biobío: Historia Natural de un Ecosistema de uso múltiple. Publicaciones Centro EULA. 2006. http://www.eula.cl/images/stories/documentos/3.pdf

[36] Bórquez J, Samollow PB, Douglas KC, Jasti M, Bertin A, Gouin N. Characterization of microsatellite loci for the Chilean freshwater gastropod *Chilina dombeiana*. (Molecular Ecology Resources Primer Development Consortium. 2011. Permanent Genetic Resources added to Molecular Ecology Resources Database 1 February 2011–31 March 2011.) Mol Ecol Resour. 2011; 11:757–758. Available from: http://biomath.trinity.edu/manuscripts/11-4/mer-11-0072.pdf 10.1111/j.1755-0998.2011.03028.x 21627775

[37] Raymond M, Rousset F. GENEPOP (Version-1.2): population genetics software for exact tests and ecumenicism. J Hered. 1995; 86: 248–249.

[38] Rice WR. Analyzing tables of statistical tests. Evolution. 1989; 43: 223–225. 28568501 10.1111/j.1558-5646.1989.tb04220.x

[39] Van Oosterhout C, Hutchinson WF, Wills DPM, Shipley P. MICROCHECKER: software for identifying and correcting genotyping errors in microsatellite data. Mol Ecol Notes. 2004; 4:535–538.

[40] Belkhir K, Borsa P, Chikhi L, Raufaste N, Bonhomme F. GENETIX 4.05, logiciel sous Windows TM pour la génétique des populations Laboratoire Génome, Populations, Interactions, CNRS UMR 5000, Université de Montpellier II, Montpellier (France); 1996–2004.

[41] Goudet J. FSTAT (V 1.2): a computer program to estimate F-statistics. J Hered. 1995; 86: 485–486.

[42] Jones AG. GERUD 2.0: a computer program for the reconstruction of parental genotypes from half-sib progeny arrays with known or unknown parents. Mol Ecol Notes. 2005; 5: 708–711.

[43] Neff BD, Pitcher TE. Assessing the statistical power of genetic analyses to detect multiple mating in fishes. J Fish Biol. 2002; 61: 739–750.

[44] Le Cam S, Riquet F, Pechenik JA, Viard F. Paternity and gregariousness in the sex-changing sessile marine gastropod *Crepidula convexa*: Comparison with other protandrous *Crepidula* species. J Hered. 2014; 105: 397–406. 10.1093/jhered/esu002 24489076

[45] Makinen T, Panova M, André C. High levels of multiple paternity in *Litorina saxatilis*: hedging the bets?. J Hered. 2007; 98: 705–711. 10.1093/jhered/esm097 18056922

[46] Wang J. Sibship reconstruction from genetic data with typing errors. Genetics. 2004; 166: 1963–1979. 15126412 10.1534/genetics.166.4.1963 PMC1470831

[47] Jones O, Wang J. COLONY: a program for parentage and sibship inference from multilocus genotype data. Mol Ecol Resour. 2010; 10: 551–555. 10.1111/j.1755-0998.2009.02787.x 21565056

[48] Karaket T, Poompuang S. CERVUS v/s COLONY for successful parentage and sibship determinations in freshwater prawn *Macrobrachium rosenbergii*. Aquaculture. 2012; 324–325: 307–311.

[49] Wang J. COANCESTRY: a program for simulating, estimating and analyzing relatedness and inbreeding coefficients. Mol Ecol Resour. 2011; 11: 141–145. 10.1111/j.1755-0998.2010.02885.x 21429111

[50] Queller DC, Goodnight KF. Estimating relatedness using genetic markers. Evolution. 1989; 43: 258–275. 28568555 10.1111/j.1558-5646.1989.tb04226.x

[51] Lynch M, Ritland K. Estimation of pairwise relatedness with molecular markers. Genetics. 1999; 152: 1753–1766. 10430599 10.1093/genetics/152.4.1753 PMC1460714

[52] Wang J. An estimator for pairwise relatedness using molecular markers. Genetics. 2002; 160: 1203–1215. 11901134 10.1093/genetics/160.3.1203 PMC1462003

[53] R Core Team. R: A Language and Environment for Statistical Computing. R Foundation for Statistical Computing, Vienna, Austria 2014 http://www.R-project.org/

[54] David P, Pujol B, Viard F, Castella V, Goudet J. Reliable selfing rate estimates from imperfect population genetic data. Mol Ecol. 2007; 16: 2474–2487. 10.1111/j.1365-294X.2007.03330.x 17561907

[55] Jarne P, Finot L, Delay B, Thaler L. Self-fertilization versus cross-fertilization in the hermaphroditic freshwater snail *Bulinus globosus*. Evolution. 1991; 45: 1136–1146. 28564176 10.1111/j.1558-5646.1991.tb04380.x

[56] Jarne P, Vianey-Liaud M, Delay B. Selfing and outcrossing in hermaphrodite freshwater gastropods (Basommatophora): where, when and why. Biol J Linn Soc Lond. 1993; 49: 99–125.

[57] Mavárez J, Amarista M, Pointier P, Jarne P. Fine-scale population structure and dispersal in *Biomphalaria glabrata*, the intermediate snail host of *Schistosoma mansoni*, in Venezuela. Mol Ecol. 2002; 11: 879–889. 11975704 10.1046/j.1365-294x.2002.01486.x

[58] Viard F, Doums C, Jarne P. Selfing, sexual polymorphism and microsatellites in the hermaphroditic freshwater snail *Bulinus truncatus*. Proc R Soc Lond B Biol Sci. 1997; 264: 39–44.

[59] Tsitrone A, Jarne P, David P. Delayed selfing and resource reallocations in relation to mate availability in the freshwater snail *Physa acuta*. Am Nat. 2003; 162: 474–488. 10.1086/376889 14582009

[60] Noël E, Chemtob Y, Janicke T, Sarda V, Pélissié B, Jarne P, David P. Reduced mate availability leads to evolution of self-fertilization and purging of inbreeding depression in a hermaphrodite. Evolution. 2016; 70: 625–640. 10.1111/evo.12886 26899922

[61] Dupont L, Richard J, Paulet YM, Thouzeau G, Viard F. Gregariousness and protandry promote reproductive insurance in the invasive gastropod *Crepidula fornicata*: evidence from assignment of larval paternity. Mol Ecol. 2006; 15: 3009–3021. 10.1111/j.1365-294X.2006.02988.x 16911217

[62] Mulvey M, Vrijenhoek RC. Multiple paternity in the hermaphroditic snail *Biomphalaria obstructa*. J Hered. 1981; 72: 308–312.

[63] Rudolph PH, Bailey JB. Copulation as females and use of allosperm in the freshwater snail genus *Bulinus*. J Molluscan Stud. 1985; 51: 267–275.

[64] Rollinson D, Kane RA, Lines JRL. An analysis of fertilization in *Bulinus cernicus* (Gastropoda: Planorbidae). J Zool. 1989; 217: 295–310.

[65] Wethington AR, Dillon RT Jr. Sperm storage and evidence for multiple insemination in a natural population of the freshwater snail, *Physa*. Am Malacolol Bull. 1991; 9: 99–102.

[66] Städler T, Weisner S, Streit B. Outcrossing rates and correlated matings in a predominantly selfing freshwater snail. Proc R Soc Lond B Biol Sci. 1995; 262: 119–125. 10.1098/rspb.1995.0185 8524906

[67] Coutellec-Vreto MA, Madec L, Guiller A. Selfing and biparental inbreeding: a mating system analysis in *Lymnaea peregra* (Gastropoda: Lymnaeidae). Heredity. 1997; 79: 277–285.

[68] Oppliger A, Naciri-Graven Y, Ribi G, Hosken DJ. Sperm length influences fertilization success during sperm competition in the snail *Viviparus ater*. Mol Ecol. 2003; 12: 485–492. 12535098 10.1046/j.1365-294x.2003.01748.x

[69] Garefalaki ME, Triantafyllidis A, Abatzopoulos TJ & Staikou A. The outcome of sperm competition is affected by behavioural and anatomical reproductive traits in a simultaneously hermaphroditic land snail. J Evol Biol. 2010; 23: 966–976. 10.1111/j.1420-9101.2010.01964.x 20298442

[70] Zahradnik TD, Lemay MA, Boulding EG. Choosy males in littorinid gastropod: male *Littorina subrotundata* prefer large and virgin females. J Molluscan Stud. 2008; 74: 245–251.

[71] Johnson SL, Brockmann HJ. Cost of multiple mates: an experimental study in horseshoe crabs. Anim Behav. 2010; 80: 773–782.

[72] Pizzari T, Wedell N. The polyandry revolution. Philos Trans R Soc Lond B Biol Sci. 2013; 368: 20120041 10.1098/rstb.2012.0041 23339233 PMC3576576

[73] Neff BD, Pitcher TE. Genetic quality and sexual selection: an integrated framework for good genes and compatible genes. Mol Ecol. 2005; 14: 19–38. 10.1111/j.1365-294X.2004.02395.x 15643948

[74] Burdfield-Steel ER, Auty S. & Shuker DM. Do benefits of polyandry scale with outbreeding?. Behav Ecol. 2015; 26: 1423–1431. 10.1093/beheco/arv103 26379413 PMC4568444

[75] Yue GH, Chang A. Molecular evidence for high frequency of multiple paternity in a freshwater shrimp species *Caridina ensifera*. PLoS ONE. 2010; 5:e12721 10.1371/journal.pone.0012721 20856862 PMC2939052

[76] Tregenza T, Wedell N. Polyandrous females avoid costs of inbreeding. Nature. 2002; 415: 71–73. 10.1038/415071a 11780118

[77] Zeh JA, Zeh DW. Outbred embryos rescue inbred half siblings in mixed paternity broods of live-bearing females. Nature. 2006; 439: 201–203. 10.1038/nature04260 16407952

[78] Nilsson C, Reidy CA, Dynesius M, Revenga C. Fragmentation and flow regulation of the world´s large river systems. Science. 2005; 308: 405–408. 10.1126/science.1107887 15831757

[79] Valdovinos C. Invertebrados dulceacuícolas In: Conama, editor. Biodiversidad de Chile. Patrimonio y desafíos. Ocho Libros Editores, Santiago de Chile; 2006 pp. 204–225.

